# Monthly Summary

**Published:** 1879-08

**Authors:** 


### MONTHLY SUMMARY.

*New Sources of Rubber.*—The director of Kew Gardens (Eng.) has given much attention to the matter of extending the sources whence this valuable product is obtained. In his annual report he states that though a large proportion of the young plants of the Para rubber (*Hevea Braziliensis*) brought to Kew failed to thrive, seeds and plants of the Ceara rubber have been obtained, and a considerable stock successfully raised. Para rubber plants have been transmitted to Calcutta for distribution to Assam and Burmah, where, it seems, they are now doing well. Favorable reports qave also been received from Singapore, where it is said that, judging from the progress the plants have made, the climate is evidently suited for their growth. The same may be said of Ceylon, whence the superintendent of the government garden reports that cuttings of *Hevea* strike readily, as well as those of *Castilloa* and the Ceara plant.

In Jamaica, also, the plants of *Hevea* are doing well. The propagation of the Central American rubber plant (*Castilloa elastica*) is still being proceeded with at Kew, and during the past year plants of this species were sent to Liberia, Mauritius, Singapore, and Ceylon. The Ceara rubber, owing to its totally different habit from that of the other two species, will, it is thought, prove to be the best fitted for cultivation in Bengal and the drier parts of India.

Regarding new sources of India rubber, reference is made to a creeping Burmese plant, the *Chavannesia esculenta*, which was first noticed so long back as 1860, and again made the subject of a pamphlet published in India in 1874. The plant is there stated to be one "for whose extermination in the teak tracts an abundant provision is made." From Fiji samples of rubber were received at Kew, which were reported as "a strong, elastic, pure rubber, of the same character as the higher grades of African rubber." This rubber would seem to be the produce of a plant closely allied to *Tabernæmontana pacifica* or from *Alstonia plumosa*, both of which appear to yield caoutchouc in Fiji, and both of which belong to the same natural order Apocynaceæ. Regarding the rubber producing plants of the east and west coasts of Africa, which are referred to as species of *Landolphia*, also belonging to the same natural family as the preceding, the director reports that, "being climbing plants which ascend lofty trees, they coul not be grown like other rubber producing trees in independent plantations. But they would doubtless flourish in the jungles of any tropical country."—*Scientific American.*

---

*Amalgams in Children's Teeth.*—After I had found the statement to be true, "that every tooth plugged with a metal is a galvanic battery," it was easy to discover that children's teeth are more "positive" than the teeth of adults. Consequently they would be put in more peril from a gold filling than would better calcified teeth. In children's mouths the battery would be more likely to be set in operation, than in adult mouths, for the reason that children's mouths oftener contain acidulous fluids than others.

Theory and experiment in this direction harmonize perfectly. Acting on my convictions, I early began to use Fletcher's Amalgam in children's teeth. This week I have examined the mouths of several children for whom I filled teeth five years ago with Fletcher's "Gold and Platinum Alloy." One was a child eight years old at the time. Another was a ten year old child. In the mouth of the latter is a sixth year molar containing a large anterior proximal, a large crown, and a large buccal cavity, filled with that Amalgam five years ago, and now well preserved. A ten year old girl had four cavities in the upper incisors; one so large as to involve the palatine wall. These were filled with the same Amalgam, and are to-day in good condition. The surfaces of these fillings are blackened, but that is of little consequence compared to their preservation. I recommend these fillings to remain until pride of appearance requires their removal. In the case of boys it might never be ; in the case of young ladies it would be demanded at 17 or 18 years of age.

I feel quite sure that Amalgams are far superior to gold for the preservation of children's teeth. In filling the *crevices* of teeth with gold foil it is necessary to open them *much wider* than it is to use Amalgams. There are other reasons for their use which will suggest themselves to the mind of the reader.

According to my experiments, the strength of a tooth battery is as follows: Gold and Tooth, 6 ; Amalgam and Tooth, 4 ; Tin and Tooth, 3 ; Hill's Stopping and Tooth, 0.

We look for a better filling than we now have. Mr. Fletcher has done so much in this line that I look to him for something far better than has yet been discovered. I am pleased with the working of his "Porcelain Cement," but I cannot yet judge of its merits. We wish for something still more insoluble in organic acids.—*Dental Advertiser.*

---

*New Experiments in Anæsthetics.*—An experiment of considerable practical interest was performed a few days ago by MM. Labbé, Bert, Preterre, Lafont, and Regnaud, for the purpose of testing the practical applications of Professor Bert s researches on the anæsthetic properties of mixed nitrous oxide and oxygen *under tension.* You are doubtless aware of the character of M. Bert's researches, which were communicated to the Academy of Science in one of its recent sittings. But the experiment to which I allude was a practical one, applied to a human being. A chamber with compressed air having been prepared, the experimenters entered it with a young woman of twenty, who was to be operated upon for that most painful operation, ingrowing nail. As soon as the barometer marked an increase of pressure equal to 0.17 centimetres, M. Preterre, the well-known dentist, applied the apparatus which he is in the habit of using. There was a sudden cessation of breathing, which lasted about fifteen seconds. Then a long inspiration followed, and after ten seconds there was complete insensibility. Dr. Labbé now proceeded quietly and leisurely with the operation, followed by the dressing. All this took about eight minutes, during which time the patient slept quietly, with a regular pulse, and a clear, rosy complexion. On waking up she immediately felt the pain, and had a sort of short, hysterical fit, with crying. But she declared when it ended that she felt quite well and very hungry, as she had not had anything yet to eat. The assistants were struck with the way in which she recovered her normal condition, as she was able to walk immediately, and to resume her habits. The value of this anæsthetic mixture of about eighty-five parts of nitrous oxide and fifteen of oxygen, administered *under tension,* and discovered by Prof. Bert, therefore promises to be very useful and practical. With this mixture, employed in compressed air, the patient does not get blue in the face, and the natural complexion, pulse, and breathing seem to be preserved. Moreover, it is not preceded by the period of agitation which often proves so tedious and troublesome, and is not followed by the stage of reaction which often upsets a patient for several consecutive hours.—*London Lancet.*

---

*Utah Mineral Wax.*—The great deposit of mineral wax, or native paraffine, lately discovered in Southern Utah, is described by Professor J. E. Clayton, of Salt Lake City, as occupying an area 60 miles long by 20 miles wide, and in some places forming a bed 20 feet thick. It contains more or less clay in seams and layers; but this is readily eliminated by melting, the earthly matter settling and leaving the paraffine nearly pure. It is quite black in the mass, but the sections are quite translucent. The quantity is said to be enormous; so great, indeed, that it cannot be controlled by any individual or company, but must prove a source of wealth to whole communities.

Professor Henry Wurtz pronounces the mineral to be zietriskisite, and says that it differs from paraffine by being insoluble in ether, and otherwise. Professor J. S. Newberry finds the specimens brought by him from Utah to be true ozocerite, and similar in all respects, except color, to that from Galicia; a true paraffine, melting at 60°, and being soluble in ether.

As to the origin and geological relations of this remarkable bed of paraffine—which so far as known, is without parallel in the world, and is as much of a "wonder" as our basins of petroleum, Professor Newberry cannot speak with any confidence until he has visited the locality where it occurs, as he hopes to do in a few weeks. He suspects, however, that it will be found to be an evolved product, the distillation of beds of cretaceous lignite, and the residue of a petroleum unusually rich in paraffine.—*Scientific American.*

---

*An Anomalous Case of Replantation.*—Dr. Harlan states the following case: A gentleman in the endeavor to open the apex of the root of an upper left lateral incisor tooth, with a fine needle, was so unfortunate as to break off the needle in his tooth, after it had passed through the foramen a distance exceeding an eighth of an inch. This accident happened on Sunday at four A. M., the 26th of May, 1878. He was sitting on my office steps when I arrived there on Monday at half-past eight A. M.

I worked an hour or more trying to dislodge the remnant of the needle, but my attempt to do so was futile. Not having more time at my disposal, he was dismissed until half-past four P. M., having previously depleted the gum. I did not know at the time that the needle had passed through the end of the root, and as he had not preserved the other portion of the needle, I was unable to determine that point. The root, as I found afterwards, was a trifle more than three-fourths of an inch in length, and that portion of the needle which had not passed through the apex, was not longer than the thirty secondth part of an inch—rendering it impossible to dislodge it in the ordinary manner. At the appointed time the gentleman returned in great agony. After a few minutes I extracted the tooth and found what has been related above. Here was a case for replantation; as the gentleman had only seven front teeth, and two molars on opposite sides, he readily agreed to my proposal to replace the tooth, which was done in somewhat less than an hour from the time it was extracted. The hour being late, I filled the crown with gutta percha, and the root with gold. Previous to the replacement of the tooth I held it in tepid water for a few moments, and also caused a free flow of blood from the socket ligating it to the adjoining teeth; I bathed the gum daily two or three times with the tincture of arnica only. By the following Monday the tooth was firm in its socket. On the last day of July I filled the crown of the tooth with gold, restoring one-fourth of it—using the mallet to consolidate the filling. There was no pain or soreness. The tooth is still in good condition.—*Dental Register.*

---

*New Thing for Cleansing Surgical Instruments.*—A French journal describes a convenient preparation for this purpose, called *stilbeine.* It is simply a combination of rubber and impalpable emery powder. It comes in tablets of the shape and size of the ordinary rubber eraser. It removes rust and blood-stains, etc., immediately, and without scratching or soiling the instrument. All that is necessary is to rub the instrument with the composition, when it rapidly acquires a brilliant polish. It ought to be introduced into this country.—*Drug. Circular.*

---

*The Use of Iodoform.*—Finely powdered iodoform, or mixed one part to three with unguentum petrolei. makes an admirable application to the most sensitive surface, such as irritable ulcers, etc. It is a good rule never to apply soap to such surfaces; even the best obtainable is often irritating; and water should be thoroughly boiled and used when cooling. To do away the odor of the iodoform, so nauseous to many, it may be mixed with equal parts of tannin, or employed in ethereal solution.—*Med. and Surg. Reporter.*

